# Efficacy and safety of ripertamab in immune-mediated glomerular diseases with proteinuria: an expanded real-world cohort study

**DOI:** 10.3389/fimmu.2026.1969321

**Published:** 2026-09-30

**Authors:** Xichao Wang, Yingying Han, Nan Hu, Jinping Li, Wenyu Zhang, Wenxiu Chang

**Affiliations:** Department of Nephrology, Tianjin First Central Hospital, Tianjin, China

**Keywords:** B-cell-targeted therapy, immune-mediated glomerular diseases, primary membranous nephropathy, proteinuria, real-world study, ripertamab

## Abstract

**Background:**

Ripertamab (SCT400), a recombinant anti-CD20 monoclonal antibody with enhanced antibody-dependent cell-mediated cytotoxicity, has shown efficacy in primary membranous nephropathy (PMN). This study evaluated its broader therapeutic potential in immune-mediated glomerular diseases with proteinuria.

**Methods:**

In this ambispective real-world cohort study, 176 adult patients with biopsy-confirmed immune-mediated glomerular diseases presenting with proteinuria were enrolled, comprising 52 patients from a prior PMN analysis and 124 newly enrolled patients with diverse pathologies. Patients were stratified into initial therapy (n = 131) and alternative therapy (n = 45, prior treatment failure or relapse) groups. The PMN cohort (n = 150) was designated as the primary efficacy population; the primary efficacy outcomes were the proportions of patients achieving complete remission (CR) or partial remission (PR) at 3, 6, 9, and 12 months in the PMN cohort. Outcomes of non-PMN patients (n = 26) were prespecified as exploratory and descriptive only.

**Results:**

The median potential follow-up was 19.5 (9.3, 28.5) months, and the median observed clinical follow-up was 9.3 (4.7, 18.1) months. In the PMN cohort, among patients with evaluable data at each respective timepoint, the CR/PR proportions were 45.6% (41/90) at 3 months, 54.4% (37/68) at 6 months, 59.6% (34/57) at 9 months, and 66.7% (30/45) at 12 months, with a 12-month CR rate of 15.6% (7/45). Previously treated patients achieved remission significantly more slowly than treatment-naive patients (median 232.5 vs. 83.0 days, P = 0.040), whereas complete-remission time did not differ; 12-month estimates were limited by small subgroups. Of the 77 PMN patients who achieved remission and had subsequent follow-up, 25 (32.5%) relapsed. Safety was favorable: AE incidence was 9.1%, with no Grade ≥3 AEs or treatment-related deaths.

**Conclusions:**

In this single-arm, observational PMN cohort, ripertamab monotherapy was associated with clinically meaningful remission and favorable tolerability; causality cannot be established without a concurrent control group. Among evaluable patients, 12-month CR/PR was 69.4% and 55.6% in treatment-naïve and previously treated patients, respectively; the latter was based on only nine patients, who also remitted more slowly. These hypothesis-generating findings warrant controlled evaluation of ripertamab in PMN, including refractory disease. Non-PMN evidence remains exploratory.

## Introduction

Primary membranous nephropathy (PMN) represents a leading cause of nephrotic-range proteinuria and immune-mediated glomerular disease in adults (1). While the therapeutic landscape for PMN has evolved substantially with the advent of B-cell-targeted therapies, a considerable proportion of patients with proteinuric immune-mediated glomerular diseases present with pathological subtypes beyond PMN, including mesangial proliferative glomerulonephritis, IgA nephropathy, focal segmental glomerulosclerosis (FSGS), and minimal change disease (2, 3). These conditions, though heterogeneous in etiology, share common immunopathological features involving B-cell-mediated dysregulation and aberrant cytokine signaling that drive disease progression (4, 5).

Rituximab (RTX), a chimeric anti-CD20 monoclonal antibody, has transformed the treatment paradigm for B-cell-driven autoimmune glomerulopathies, achieving remission rates of 60% to 80% in PMN (6, 7). The Kidney Disease: Improving Global Outcomes (KDIGO) 2021 guideline recommends RTX as a first-line immunosuppressive option for PMN patients with progressive disease risk factors (8). Beyond PMN, emerging evidence supports the efficacy of RTX in refractory FSGS and other immune-mediated glomerulopathies through broad-spectrum B-cell depletion and modulation of downstream inflammatory cascades (9, 10). Nevertheless, a substantial proportion of patients fail to achieve remission or experience relapse following RTX therapy, and the optimal B-cell-targeted agent for diverse glomerular pathologies remains to be defined.

Ripertamab (SCT400) is a recombinant human-mouse chimeric anti-CD20 immunoglobulin G1 (IgG1) monoclonal antibody that shares the same antigen-binding variable region as RTX, with a single amino acid substitution at position 219 of the heavy chain constant region 1 (11). Preclinical characterization has demonstrated that this structural modification results in optimized glycosylation and enhanced binding affinity to FcγRIIIa receptors on effector cells, thereby augmenting antibody-dependent cell-mediated cytotoxicity (ADCC) activity compared with the originator molecule (11, 12). These pharmacological properties suggest a potentially superior therapeutic index for B-cell depletion in autoimmune indications. Ripertamab was approved for CD20-positive B-cell non-Hodgkin lymphoma in 2022, and subsequent clinical studies have established comparable pharmacokinetics, pharmacodynamics, and safety profiles to RTX in oncology populations (11, 12). In the context of glomerular diseases, B-cell depletion by anti-CD20 therapy has been shown to reduce pathogenic autoantibody production, attenuate complement activation, and restore immune tolerance in both membranous nephropathy and other immune-mediated glomerulopathies (13–15). Given that ripertamab exhibits enhanced ADCC activity compared with RTX, it is plausible that this pharmacological advantage may translate into more efficient B-cell depletion within the glomerular microenvironment, potentially accelerating the reduction of pathogenic autoantibody titers. However, no direct comparative data in autoimmune nephropathy models are currently available, and extrapolation from oncology pharmacodynamics to glomerular disease remains speculative.

We previously conducted a multicenter retrospective analysis of 52 PMN patients receiving ripertamab monotherapy (39 treatment-naïve and 13 with prior immunosuppressive treatment failure or relapse), reporting a clinical remission rate of 60.0% at 6 months and 77.3% at 12 months, with favorable tolerability (16). Although encouraging, that study was limited by its small sample size, short follow-up (median 8.7 months), and only 13 previously treated patients, precluding reliable conclusions on the effect of prior treatment exposure. The efficacy of ripertamab in patients with prior immunosuppressive exposure and in non-PMN glomerular pathologies remains unclear.

The present study was designed to address these knowledge gaps through an expanded ambispective cohort. By integrating extended follow-up of patients from our prior study with newly enrolled participants across diverse immune-mediated glomerular diseases and proteinuria subtypes, we sought to evaluate the efficacy and safety profile of ripertamab in a broader clinical context. Patients were stratified into an initial therapy group - those without prior immunosuppressive exposure-and an alternative therapy group comprising individuals who had experienced treatment failure or relapse following previous immunosuppressive regimens, across the spectrum of biopsy-proven immune-mediated glomerular pathologies, with exploratory assessment of non-PMN subtypes. This expanded investigation aims to delineate the therapeutic potential of ripertamab across the spectrum of immune-mediated glomerular diseases with proteinuria and to substantiate its role as a B-cell-targeted intervention in real-world clinical practice.

## Materials and methods

### Study population

This ambispective real-world cohort study was conducted across three tertiary nephrology centers in Tianjin. The study protocol was approved by the institutional review board of Tianjin First Central Hospital (No. 20240423-1), and all data collection, storage, and analysis were conducted in accordance with the guidelines for using real-world data to generate real-world evidence, as outlined by the Center for Drug Evaluation in China.

### Study design and patient cohort integration

This study is an ambispective real-world cohort study: data of the previously published cohort (from January 2023) were collected retrospectively from electronic medical records, whereas patients in the expansion phase were prospectively enrolled into a unified multicenter real-world registry (through May 2026) using standardized case report forms and prespecified follow-up timepoints. The present analysis was performed retrospectively after enrollment closure and completion of follow-up. An interim analysis of the first 52 consecutive patients with biopsy-confirmed primary membranous nephropathy (PMN)-enrolled from January 2023 to October 2024 and followed for a median of 8.7 [4.7, 11.3] months-was previously published (16). The present study reports the complete cohort findings, incorporating the full follow-up extension of these 52 original patients (median observed follow-up now 18.8 [12.9, 28.5] months) together with 124 subsequently enrolled patients treated between January 2023 and May 2026, yielding a total analytic sample of 176 adult patients with immune-mediated glomerular diseases and proteinuria.

The expanded cohort design serves two primary purposes. First, it enables longitudinal assessment of long-term efficacy and safety outcomes in the original PMN population through substantially extended observation. Second, it broadens the generalizability of our findings by extending inclusion criteria to encompass patients with diverse immune-mediated glomerular pathologies beyond PMN, including mesangial proliferative glomerulonephritis, IgA nephropathy with nephrotic-range proteinuria, focal segmental glomerulosclerosis (FSGS), and minimal change disease. This expansion was motivated by the shared immunopathological features of B-cell-mediated dysregulation and aberrant cytokine signaling across these heterogeneous conditions (4, 5), as well as by emerging evidence supporting the efficacy of anti-CD20 therapy in refractory non-PMN glomerulopathies (9, 10).

It is important to note that the inclusion criteria differed between the two study phases: the original interim analysis was restricted to biopsy-confirmed PMN patients receiving monotherapy, whereas the present expansion broadened eligibility to encompass diverse immune-mediated glomerular pathologies with nephrotic presentation, including patients with prior immunosuppressive exposure.

### Inclusion criteria

Patients were eligible for inclusion if they met the following criteria: (i) adult patients (≥18 years) with biopsy-confirmed immune-mediated glomerular diseases including PMN, mesangial proliferative glomerulonephritis, IgA nephropathy, FSGS, or minimal change disease; (ii) patients who received at least one complete course of ripertamab monotherapy, administered as either (a) intravenous 375 mg/m² once weekly for 4 weeks, or (b) 1 g per dose every two weeks for a total of 2 doses; and (iii) patients who initiated ripertamab therapy between January 2023 and May 2026. For the 52 patients included in the prior interim analysis (16), baseline and follow-up data from the original enrollment period were retained, with additional longitudinal data collected at 6-month intervals through May 2026 to outcomes.

### Exclusion criteria

Patients were excluded if they had: (i) secondary glomerular diseases including lupus nephritis, Henoch-Schönlein purpura nephritis, or diabetic nephropathy; (ii) receipt of rituximab or its biosimilars within three months before enrollment or during the treatment period; (iii) concurrent use of glucocorticoids, calcineurin inhibitors, alkylating agents, or other immunosuppressive agents during ripertamab therapy; (iv) participation in other interventional clinical trials during the treatment period; or (v) a history of malignant tumors or organ transplantation.

### Group stratification

Based on prior immunosuppressive exposure before ripertamab initiation, patients were categorized into two groups: the initial therapy group (n = 131), defined as patients who had not received any prior immunosuppressive treatment for proteinuria; and the alternative therapy group (n = 45), defined as patients who had experienced treatment failure or relapse following previous immunosuppressive regimens (including glucocorticoids, calcineurin inhibitors, cyclophosphamide, or mycophenolate mofetil). Among the 52 patients from the interim analysis, 39 were classified into the initial therapy group and 13 into the alternative therapy group, consistent with the prior publication (16), in that the treatment-naïve and retreatment groups of the prior study correspond to the initial-therapy and alternative-therapy groups of the present study, respectively; no reclassification occurred. The 124 additional patients comprised 92 in the initial therapy group and 32 in the alternative therapy group.

### Diagnostic confirmation and pathological classification

Immune-mediated glomerular disease was diagnosed based on renal biopsy findings combined with clinical presentation, serological testing, and systematic exclusion of secondary etiologies. All biopsies were reviewed by at least one experienced renal pathologist at the participating center, with immunofluorescence microscopy and electron microscopy performed in all cases according to standard protocols.

Primary membranous nephropathy was defined by subepithelial immune complex deposition with immunofluorescence showing granular IgG and C3 staining along capillary walls, and electron microscopy confirming subepithelial electron-dense deposits. Serum anti-PLA2R antibodies were measured by indirect immunofluorescence assay (IIFT), with a titer of ≥1:10 defined as positive; no RU/mL quantitative data were available in this study. Testing was ordered by treating physicians as clinically indicated, and the baseline testing rate and retesting results are reported transparently in the Results. Anti-PLA2R titers (when available), together with 24-hour urinary protein and serum albumin, informed retreatment decisions. Anti-PLA2R-negative cases were retained as primary membranous nephropathy if biopsy features were classical and secondary causes were excluded.

Non-PMN glomerulopathies were classified as follows: Mesangial proliferative glomerulonephritis was defined by mesangial hypercellularity with or without endocapillary proliferation; IgA nephropathy was diagnosed by dominant mesangial IgA deposition on immunofluorescence with electron-dense deposits in mesangial regions; minimal change disease by diffuse foot process effacement on electron microscopy with negative immunofluorescence; and FSGS by segmental glomerular scarring with podocyte foot process effacement.

Quality assurance. A random subset of 30 biopsies, 15 primary membranous nephropathy and 15 non-PMN, was independently reviewed by a single reference renal pathologist blinded to the original diagnosis and clinical outcomes. Inter-observer agreement was substantial, with Cohen’s kappa of 0.82, 95% confidence interval 0.71 to 0.93, for primary diagnosis category, and kappa of 0.76, 95% confidence interval 0.62 to 0.90, for primary membranous nephropathy versus non-PMN distinction.

Reclassification protocol. One patient initially coded as diabetic nephropathy was reclassified as primary membranous nephropathy with concurrent diabetes mellitus after blinded reference review confirmed characteristic subepithelial deposits with negative PLA2R but positive THSD7A staining. This case illustrates the importance of biopsy re-evaluation and was retained as primary membranous nephropathy in all analyses.

### Treatment and follow-up

In the real-world setting, ripertamab was administered according to two established regimens: (1) intravenous infusion at 375 mg/m² once weekly for 4 consecutive weeks, or (2) 1 g per dose every two weeks for a total of 2 doses. To minimize infusion-related reactions, all patients received prophylactic premedication comprising intravenous methylprednisolone (40 mg) and oral cetirizine (10 mg) approximately 30 minutes prior to each infusion. The infusion rate was carefully titrated and monitored according to institutional protocols.

The decision to administer reinfusion was individualized based on clinical and serological assessments at the 6-month follow-up visit. Patients with persistent nephrotic-range proteinuria, elevated anti-PLA2R antibody titers, or incomplete B-cell depletion were considered for additional ripertamab doses (375 mg/m² × 1–2 doses or 1 g/dose × 1–2 doses). However, owing to constraints in testing availability and associated costs in real-world practice, routine monitoring of B-cell counts and anti-PLA2R antibody levels was not uniformly performed across all centers, and retreatment decisions were often guided by clinical remission status and physician judgment.

Demographic characteristics, treatment details, and laboratory data were extracted from the electronic medical record databases of each participating center. Follow-up assessments were scheduled at every month after ripertamab initiation. For the 52 patients from the prior interim analysis (16), original follow-up data were retained, and additional longitudinal assessments were conducted at 6-month intervals through May 2026 to capture extended outcomes. The follow-up endpoints were defined as ESRD, loss to follow-up, or death.

Laboratory parameters, including 24-hour urinary protein (UP), serum albumin, serum creatinine, and estimated glomerular filtration rate (eGFR), were assessed at baseline and at every scheduled follow-up visit. Adverse events (AEs) related to ripertamab were evaluated during infusions and throughout the entire follow-up period, with severity graded according to the Common Terminology Criteria for Adverse Events (CTCAE) version 5.0.

We report two follow-up metrics: (i) potential follow-up, defined as the interval from the first ripertamab dose to the data cutoff date (May 7, 2026); and (ii) observed clinical follow-up, defined as the interval from the first dose to the last documented clinical contact of any kind (outpatient visit, telephone follow-up, or any laboratory test). Protocol-defined efficacy evaluability is separately defined as having the complete protocol-specified laboratory panel (24-hour urinary protein, serum albumin, and serum creatinine) within each timepoint window (± 30 days); a patient may remain in clinical follow-up without meeting protocol evaluability (e.g., followed at a referral hospital without a 24-hour urinary protein measurement), and the two are reported separately.

### Outcomes

Because the definitions of complete and partial remission are derived from the KDIGO Clinical Practice Guideline for Glomerular Diseases and their prognostic significance is disease-specific, and because B-cell depletion has the clearest biological rationale in PMN, the PMN cohort (n = 150) was designated as the primary efficacy population. The full cohort (n = 176) was used for baseline characteristics, patient flow, and safety analyses; outcomes of non-PMN patients (n = 26) were prespecified as exploratory and descriptive only and are hypothesis-generating.

#### Primary outcomes

The primary efficacy outcomes were the proportions of patients achieving complete remission (CR) or partial remission (PR) at 3, 6, 9, and 12 months after ripertamab initiation in the PMN cohort. Remission was defined according to the 2021 KDIGO Clinical Practice Guideline for the Management of Glomerular Diseases (8): (1) CR was defined as 24-hour UP ≤0.3 g/d with normal kidney function (eGFR ≥60 mL/min/1.73 m² or stable creatinine); (2) PR was defined as a reduction in proteinuria of more than 50% from baseline with a 24-hour UP value <3.5 g/d and normal kidney function.

#### Secondary outcomes

Secondary outcomes included: (i) time to remission, defined as the interval from the first ripertamab injection to the first documentation of CR or PR; (ii) relapse, defined as a 24-hour UP >3.5 g/d during follow-up in patients who had previously achieved CR or PR. The relapse rate was calculated only within the at-risk population-patients who had ever achieved CR/PR and had at least one follow-up with a 24-hour UP measurement after their first documented remission; time at risk was counted from the first documented remission. Patients without post-remission follow-up had no time at risk of relapse and were not counted in the denominator; their number is reported transparently; (iii) changes in laboratory indices (24-hour UP, serum albumin, and eGFR) from baseline to each follow-up time point; and (iv) incidence and spectrum of AEs.

### Statistical analysis

Data analysis was performed using SAS version 9.4 (SAS Institute, Cary, NC, USA) and R version 4.3.1 (R Foundation for Statistical Computing, Vienna, Austria). A two-sided P-value <0.05 was considered statistically significant.

#### Descriptive statistics

Continuous variables were summarized using means with standard deviations (SD) or medians with interquartile ranges (IQRs) depending on distribution normality assessed by the Shapiro-Wilk test. Categorical variables were presented as frequencies and percentages, with missing data excluded from percentage calculations.

#### Comparative analyses

Baseline characteristics were compared between the initial therapy group and the alternative therapy group using the Mann-Whitney U test for continuous variables and the chi-square test or Fisher’s exact test for categorical variables, as appropriate. Because the initial-therapy and alternative-therapy groups differed significantly in pathological composition (PMN 93.9% vs. 60.0%, P < 0.001), baseline eGFR (P = 0.022), serum creatinine (P = 0.013), and serum albumin (P = 0.002), between-group comparisons in the full cohort are presented descriptively only, and no causal interpretation regarding the effect of prior immunosuppressive exposure is made; the effect of prior treatment status on efficacy was evaluated within the pathologically homogeneous PMN cohort. Longitudinal changes in clinical parameters (24-hour UP, serum albumin, and eGFR) from baseline to various follow-up time points were analyzed using the Mixed Model for Repeated Measures (MMRM) to evaluate both within-group and between-group longitudinal differences. The Kaplan-Meier method was used to estimate cumulative remission rates for complete remission and partial remission, and differences between groups were compared using the log-rank test.

### Sensitivity analyses

To assess the reproducibility and independence of the principal efficacy findings, a sensitivity analysis excluding the 52 previously published patients was prespecified, repeating the primary efficacy analyses (timepoint-specific CR/PR, time to remission, and relapse rate) in the 98 newly enrolled PMN patients only, presented side by side with the previously published cohort and the full PMN cohort (**Supplementary Table S4**).

### Handling of loss to follow-up

Missing data were handled using complete-case analysis for the primary outcomes. For longitudinal laboratory parameters, mixed-effects models inherently accommodate incomplete data under the assumption of missing at random.

Given the real-world design, loss to follow-up was anticipated and predefined as a key methodological challenge. At each scheduled assessment point, patients were classified into three mutually exclusive categories: (i) completed assessment, defined as outcome data (24-hour UP, serum albumin, and eGFR) available within 30 days before or after the scheduled visit date; (ii) administratively censored, encompassing death, end-stage renal disease, or transfer to a non-participating institution with verified outcome through telephone follow-up or medical record linkage; and (iii) lost to follow-up, defined as no visit documentation at 90 days or more beyond the scheduled window and no verified outcome through active tracing efforts.

To evaluate the robustness of remission estimates under potential informative censoring, we compared the remission status of patients lost to follow-up at their last evaluable timepoint before attrition with that of evaluable patients, and we conducted extreme-scenario analyses for the 12-month CR/PR rate: a worst-case scenario assuming that all unevaluable patients failed to achieve remission, and a best-case scenario assuming that all of them achieved remission. In addition, inverse probability of censoring weighting (IPCW) was performed as a sensitivity analysis: the probability of remaining under follow-up at each timepoint was estimated using a pooled logistic model including assessment timepoint, age, sex, treatment group, baseline eGFR, baseline 24-hour UP, baseline serum albumin, and remission status at the preceding timepoint; stabilized weights were computed using a model including only the assessment timepoint as the numerator (assuming monotonic censoring), were accumulated across timepoints, and were not truncated; confidence intervals were estimated by the percentile method from 1,000 patient-level bootstrap resamples. Coefficients of the censoring model and weight diagnostics are provided in **Supplementary Tables S2** and **S3**. We further emphasize that remission proportions reported at discrete timepoints (**Table 1**) are cross-sectional complete-case estimates with differing denominators and should not be interpreted as longitudinal rates within a fixed cohort. Longitudinal remission kinetics are assessed using Kaplan-Meier time-to-event methods, which appropriately account for varying follow-up durations and censoring.

**Table 1:** Remission rates, time to remission, and relapse in the PMN cohort (n = 150) treated with ripertamab.

Study timepoints	Overall (n=150)	Initial therapy (n=123)	Alternative therapy (n=27)
Complete remission rate, n/N (%)			
3 months	2/90 (2.2%)	1/78 (1.3%)	1/12 (8.3%)
6 months	4/68 (5.9%)	4/55 (7.3%)	0/13 (0.0%)
9 months	7/57 (12.3%)	6/43 (14.0%)	1/14 (7.1%)
12 months	7/45 (15.6%)	6/36 (16.7%)	1/9 (11.1%)
Clinical remission (CR/PR) rate, n/N (%)			
3 months	41/90 (45.6%)	38/78 (48.7%)	3/12 (25.0%)
6 months	37/68 (54.4%)	33/55 (60.0%)	4/13 (30.8%)
9 months	34/57 (59.6%)	28/43 (65.1%)	6/14 (42.9%)
12 months	30/45 (66.7%)	25/36 (69.4%)	5/9 (55.6%)
Time to remission (days), median [IQR]			
Complete remission	352.00 [156.50;567.00]	353.00 [154.00;498.00]	335.50 [198.00;577.75]
Clinical remission (CR/PR)	104.00 [39.00;219.50]	83.00 [36.00;163.00]	232.50 [95.50;373.50]
Relapse rate, n/N (%)			
Relapse	25/77 (32.5%)	20/63 (31.7%)	5/14 (35.7%)

CR, complete remission; PR, partial remission. The denominator at each timepoint is the number of patients with the complete protocol-specified laboratory panel (24-hour UP, serum albumin, and serum creatinine) within that window (± 30 days) and differs across timepoints; these are cross-sectional complete-case estimates and should not be interpreted as longitudinal trajectories within a fixed cohort. Time to remission was calculated from the first ripertamab dose to the first documentation of CR or PR (the median time to PR alone in the alternative-therapy group was 248.0 days; see **Supplementary Table S4**). Relapse was defined as a 24-hour UP >3.5 g/d in patients who had previously achieved CR or PR; the denominator of the relapse rate is patients who achieved remission and had follow-up with a 24-hour UP measurement thereafter (at-risk population: 77 overall, 63 in the initial-therapy group, and 14 in the alternative-therapy group), with time at risk counted from the first documented remission; patients without post-remission follow-up (n = 18) were not counted in the denominator.

## Results

### Baseline characteristics

A total of 176 adult patients with immune-mediated glomerular diseases and proteinuria were ultimately included for analysis after screening participants against the inclusion and exclusion criteria (**Figure 1**). 131 patients received ripertamab as their initial therapy, whereas 45 patients were treated with ripertamab after experiencing a relapse or failure of prior immunosuppressive regimens (alternative therapy group). Among the 52 patients from the prior interim analysis (16), 39 were classified into the initial therapy group and 13 into the alternative therapy group.

**Figure 1:**
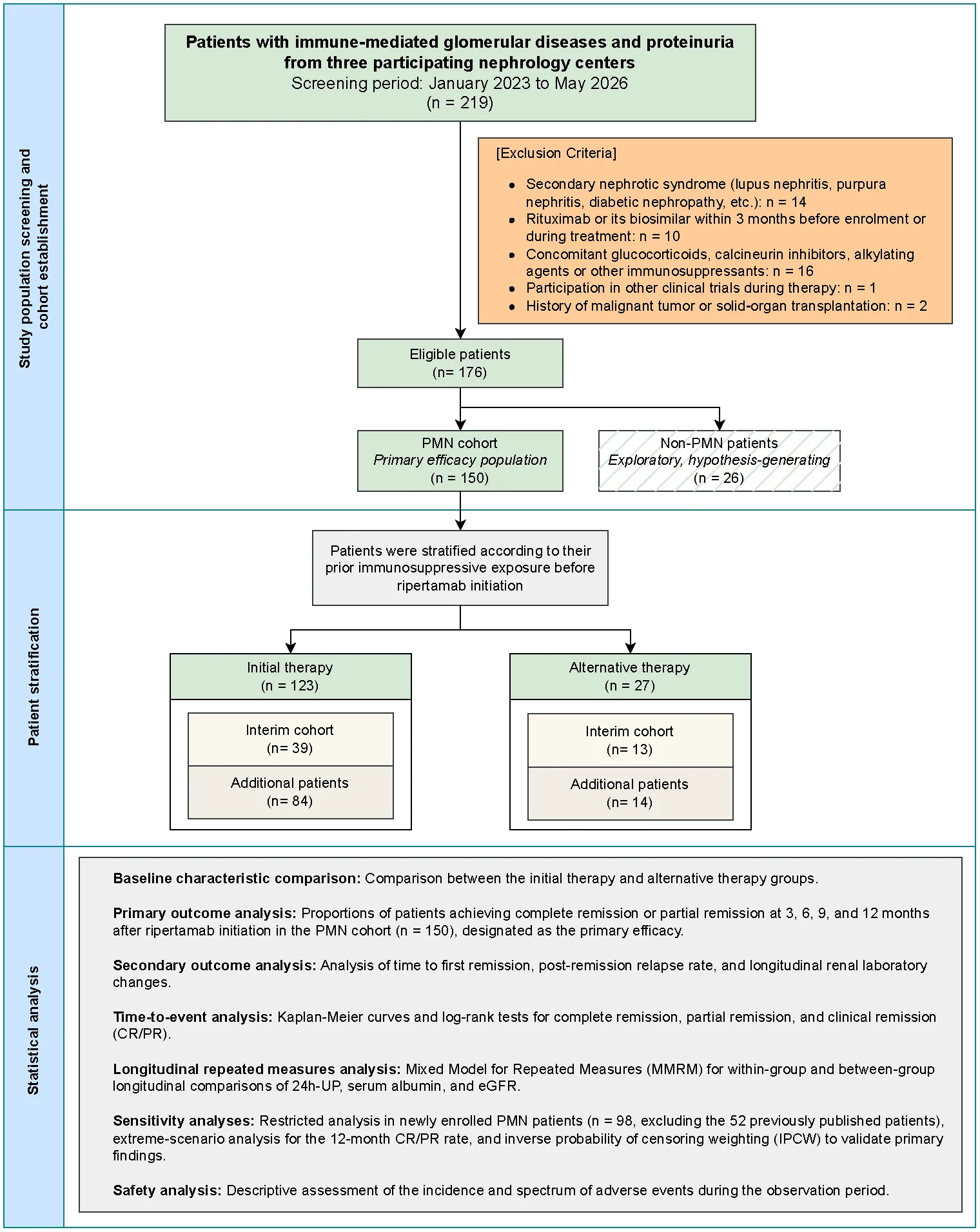
Flow chart of the patient selection process and 12-month follow-up completeness in the primary efficacy (PMN) population, distinguishing patients who had not yet reached the 12-month follow-up window (administrative censoring) from those who reached the window but had missing data or were lost to follow-up. PMN, primary membranous nephropathy; FSGS, focal segmental glomerulosclerosis; MCD, minimal change disease; MesPGN, mesangial proliferative glomerulonephritis; IgAN, IgA nephropathy.

The baseline characteristics of the study cohort are summarized in **Table 2**. The cohort was predominantly male (75.0%), with a median age of 54.0 (44.0, 62.0) years. Most patients were nondiabetic (83.5%) and had hypertension (50.0%). The median baseline 24-hour UP level was 5.96 (3.72, 9.08) g/d, the median serum albumin level was 27.10 (22.58, 32.82) g/L, the median serum creatinine level was 77.40 (66.20, 92.95) μmol/L, and the median eGFR was 94.62 (76.20, 107.25) mL/min/1.73 m².

**Table 2:** Baseline characteristics of patients with immune-mediated glomerular diseases and proteinuria receiving ripertamab.

Characteristic	Total (N = 176)	Initial therapy (N = 131)	Alternative therapy (N = 45)	P-value
Male, n (%)	132 (75.0%)	96 (73.28%)	36 (80.0%)	0.485
Age (year)	54.00 [42.00;62.25]	54.00 [40.50;62.00]	55.00 [46.00;65.00]	0.570
Smoking, n (%)	81 (46.02%)	58 (44.27%)	23 (51.11%)	0.535
Drinking, n (%)	64 (36.36%)	46 (35.11%)	18 (40.00%)	0.683
Diabetes, n (%)	29 (16.48%)	24 (18.32%)	5 (11.11%)	0.372
Hypertension, n (%)	88 (50.00%)	71 (54.20%)	17 (37.78%)	0.084
Systolic BP (mmHg)	125.00 [116.00;132.00]	125.00 [116.00;132.00]	125.00 [117.00;132.00]	0.677
Diastolic BP (mmHg)	80.00 [77.00;87.00]	80.00 [77.00;88.50]	80.00 [77.00;85.00]	0.769
BMI (kg/m²)	25.53 [23.24;28.03]	25.39 [23.22;27.92]	25.78 [23.36;28.07]	0.827
Pathological type, n (%)				<0.001
PMN	150 (85.23%)	123 (93.89%)	27 (60.00%)	
Non-PMN	26 (14.77%)	8 (6.11%)	18 (40.00%)	
Anti-PLA2R tested among PMN patients, n/N (%)	119/150 (79.3%)	94/123 (76.4%)	25/27 (92.6%)	0.069
Anti-PLA2R positive among tested, n/N (%)	81/119 (68.1%)	67/94 (71.3%)	14/25 (56.0%)	0.156
24-h UP (g/d)	5.96 [3.72;9.08]	6.34 [4.15;9.09]	5.13 [2.94;8.90]	0.089
Albumin (g/L)	27.10 [22.58;32.82]	26.30 [22.30;31.25]	31.20 [23.80;35.60]	0.002
Serum creatinine (μmol/L)	77.40 [66.20;92.95]	74.90 [65.65;88.75]	85.40 [67.60;117.60]	0.013
eGFR (mL/min/1.73m²)	94.62 [76.20;107.25]	95.68 [79.17;107.84]	85.46 [55.82;103.31]	0.022
White blood cell (×10^9^/L)	7.25 [5.87;8.41]	7.07 [5.81;7.93]	8.26 [6.03;10.54]	0.008
Platelet (×10^9^/L)	261.50 [224.25;297.50]	262.00 [223.50;297.00]	255.00 [225.00;299.00]	0.973
Total protein (g/L)	49.95 [43.00;56.00]	48.40 [42.95;54.00]	54.70 [45.40;64.30]	0.001
Globulin (g/L)	20.90 [18.78;23.62]	20.40 [18.50;23.30]	22.60 [19.50;24.50]	0.079
AST (U/L)	18.90 [15.60;23.20]	19.15 [15.60;23.05]	17.60 [15.70;24.00]	0.943
ALT (U/L)	16.10 [12.40;22.30]	15.80 [12.50;21.17]	18.90 [11.90;27.80]	0.122

ALT, alanine aminotransferase; Anti-PLA2R, anti-phospholipase A2 receptor; AST, aspartate transaminase; BMI, body mass index; BP, blood pressure; eGFR, estimated glomerular filtration rate; PMN, primary membranous nephropathy; UP, urinary protein; WBC, white blood cell.

Values are presented as numbers (%) or medians (interquartile range). Serum anti-PLA2R antibodies were measured by indirect immunofluorescence assay (IIFT; titer ≥1:10 defined as positive) in patients with PMN; denominators for the anti-PLA2R rows are PMN patients (tested row) and tested patients (positive row), respectively. eGFR was calculated according to the Chronic Kidney Disease Epidemiology Collaboration (CKD-EPI) equation. Statistical comparisons between the initial therapy group and the alternative therapy group were performed using the Mann–Whitney U test for continuous variables and the chi-square test or Fisher’s exact test for categorical variables. The initial-therapy and alternative-therapy groups differed significantly in pathological composition, baseline eGFR, serum creatinine, and serum albumin; between-group comparisons in the full cohort are confounded by these factors, and observed differences may reflect disease-specific response patterns rather than effects of prior treatment. The effect of prior treatment status is evaluated within the PMN cohort (see **Table 3** and **1**).

The non-PMN subgroup (n = 26) comprised mesangial proliferative glomerulonephritis (n = 18), IgA nephropathy with nephrotic presentation (n = 4), minimal change disease (n = 3), and focal segmental glomerulosclerosis (n = 1).

A striking difference in histopathological distribution was observed between the two groups. The initial therapy group was composed predominantly of PMN (93.89%), whereas the alternative therapy group exhibited substantial heterogeneity, with only 60.0% having PMN (P < 0.001). Additionally, the alternative therapy group exhibited lower eGFR [85.46 (55.82, 103.31) vs. 95.68 (79.17, 107.84) mL/min/1.73 m², P = 0.022], higher serum creatinine [85.40 (67.60, 117.60) vs. 74.90 (65.65, 88.75) μmol/L, P = 0.013], higher serum albumin [31.20 (23.80, 35.60) vs. 26.30 (22.30, 31.25) g/L, P = 0.002], higher white blood cell count [8.26 (6.03, 10.54) vs. 7.07 (5.81, 7.93) × 10^9^/L, P = 0.008]. These differences reflect the distinct clinical profiles of treatment-naïve versus previously treated patients, with the alternative therapy group representing a more refractory and heterogeneous population.

Of 176 enrolled patients, 105 (59.7%) completed the 3-month assessment, 80 (45.5%) at 6 months, 63 (35.8%) at 9 months, and 52 (29.5%) at 12 months. The primary reason for non-completion was transition to local follow-up after clinical stabilization (89 patients, 50.6%), reflecting standard practice in China’s tiered healthcare system where stable patients are transferred from tertiary to community hospitals. Other reasons included relocation (18 patients, 10.2%), preference for traditional Chinese medicine institutions (12 patients, 6.8%), and undocumented reasons (5 patients, 2.8%). No deaths or end-stage renal disease events occurred among lost-to-follow-up patients prior to their last documented visit.

In the primary efficacy population (PMN cohort, n = 150), the median potential follow-up was 19.5 (9.3, 28.5) months (29.8 months in the previously published cohort and 11.3 months in newly enrolled patients), and the median observed clinical follow-up was 9.3 (4.7, 18.1) months (18.8 and 7.1 months, respectively). The numbers of patients meeting protocol evaluability at 3, 6, 9, and 12 months were 90 (60.0%), 68 (45.3%), 57 (38.0%), and 45 (30.0%), respectively. The low 12-month evaluability is explained by two main factors: newly enrolled patients were enrolled late, and their median potential follow-up (11.3 months) had not yet reached the 12-month assessment window (administrative censoring); in addition, 16 patients had clinical follow-up beyond 12 months but assessments falling outside the protocol windows. The characteristics and impact of true loss to follow-up are detailed in the attrition analyses (**Table 3**, comparison by 12-month evaluability status; **Supplementary Tables S2** and **S3**).

**Table 3:** Baseline characteristics of the PMN cohort (n = 150), by treatment group and by 12-month evaluability status.

Characteristic	Overall (N = 150)	Initial therapy (N = 123)	Alternative therapy (N = 27)	P-value^a^	Evaluable at 12 months (n=45)	Unevaluable (n=105)	P-value^b^
Male, n (%)	110 (73.33%)	88 (71.54%)	22 (81.48%)	0.414	34 (75.6%)	76 (72.4%)	0.840
Age (years)	55.00 [45.25;62.75]	54.00 [43.00;62.00]	58.00 [51.00;64.50]	0.120	52.0 [41.0;60.0]	57.0 [47.0;63.0]	0.039
Smoking, n (%)	66 (44.00%)	53 (43.09%)	13 (48.15%)	0.791	15 (33.3%)	51 (48.6%)	0.123
Drinking, n (%)	56 (37.33%)	43 (34.96%)	13 (48.15%)	0.288	16 (35.6%)	40 (38.1%)	0.912
Diabetes, n (%)	25 (16.67%)	23 (18.70%)	2 (7.41%)	0.252	8 (17.8%)	17 (16.2%)	1.000
Hypertension, n (%)	78 (52.00%)	69 (56.10%)	9 (33.33%)	0.053	20 (44.4%)	58 (55.2%)	0.301
Systolic BP (mmHg)	125.00 [118.25;135.00]	125.00 [116.50;136.00]	125.00 [120.00;135.00]	0.436	124.0 [120.0;130.0]	125.0 [117.0;138.0]	0.349
Diastolic BP (mmHg)	80.00 [78.00;88.00]	80.00 [78.00;89.00]	80.00 [78.50;85.00]	0.719	80.0 [78.0;85.0]	80.0 [78.0;89.0]	0.497
BMI (kg/m²)	25.76 [23.44;28.22]	25.46 [23.35;27.92]	26.67 [24.76;29.40]	0.119	27.0 [23.9;29.3]	25.5 [23.3;27.7] (n=98)	0.090
Alternative-therapy group, n (%)	--	--	--	--	9 (20.0%)	18 (17.1%)	0.853
Previously published cohort, n (%)	--	--	--	--	33 (73.3%)	19 (18.1%)	<0.001
Anti-PLA2R tested, n/N (%)	119/150 (79.3%)	94/123 (76.4%)	25/27 (92.6%)	0.069	29/45 (64.4%)	90/105 (85.7%)	0.007
Anti-PLA2R positive among tested, n/N (%)	81/119 (68.1%)	67/94 (71.3%)	14/25 (56.0%)	0.156	18/29 (62.1%)	63/90 (70.0%)	0.494
24-h UP (g/d)	6.04 [4.05;9.09]	6.34 [4.34;9.09]	5.13 [3.21;9.12]	0.265	7.0 [5.5;9.6]	5.6 [3.6;9.1]	0.061
Albumin (g/L)	26.70 [22.25;32.05]	26.10 [22.15;31.00]	31.00 [23.30;35.00]	0.033	25.4 [22.2;30.8]	27.0 [22.4;32.2]	0.529
Serum creatinine (μmol/L)	77.40 [66.25;93.05]	76.10 [66.20;89.20]	85.40 [66.40;112.80]	0.087	81.4 [68.5;92.9]	74.2 [66.1;93.4]	0.321
eGFR (mL/min/1.73m²)	93.17 [76.31;105.00]	95.26 [78.88;106.32]	82.02 [57.45;98.35]	0.016	92.2 [77.2;103.1]	93.5 [76.2;107.2]	0.918
White blood cell (×10^9^/L)	7.08 [5.81;7.98]	7.07 [5.81;7.86]	7.34 [5.90;8.78]	0.348	6.5 [5.7;7.7]	7.1 [6.0;8.3]	0.222
Platelet (×10^9^/L)	260.00 [221.00;292.75]	261.00 [222.00;297.00]	245.00 [216.50;277.00]	0.161	263.0 [245.0;293.0]	251.0 [216.0;291.0]	0.137
Total protein (g/L)	49.20 [42.70;55.20]	48.60 [42.80;54.00]	54.20 [43.65;60.20]	0.066	49.0 [43.0;54.2]	49.7 [42.2;55.4] (n=98)	0.821
Globulin (g/L)	20.90 [18.75;23.50]	20.50 [18.70;23.35]	21.90 [19.45;24.40]	0.279	21.2 [18.9;23.5]	20.6 [18.7;23.4] (n=98)	0.503
AST (U/L)	19.40 [15.80;23.60]	19.45 [15.80;23.17]	19.30 [15.85;25.80]	0.525	19.4 [15.8;25.3]	19.4 [15.8;22.8] (n=104)	0.492
ALT (U/L)	16.00 [12.80;21.90]	15.80 [12.80;21.17]	19.40 [12.85;28.15]	0.121	16.5 [13.5;25.8]	15.9 [12.4;21.0] (n=104)	0.287

Values are presented as numbers (%) or medians [interquartile range]. Continuous variables were compared using the Mann–Whitney U test and categorical variables using the chi-square test or Fisher’s exact test.

^a^ Comparison by treatment group (initial vs. alternative therapy).

^b^ Comparison by 12-month evaluability status (evaluable vs. unevaluable). “--” indicates items applicable only to the evaluability comparison. By treatment group, residual baseline differences (eGFR, P = 0.016; serum albumin, P = 0.033) are transparently presented and acknowledged in the Discussion. Serum anti-PLA2R antibodies were measured by indirect immunofluorescence assay (IIFT; titer ≥1:10 defined as positive); among positive patients, the median first positive titer was 1:32 [IQR 1:10–1:32]. By 12-month evaluability status, evaluable patients were younger than unevaluable patients (P = 0.039), consistent with age being the only significant predictor of attrition in the censoring model (**Supplementary Table S2**), and this difference was adjusted for in the IPCW analysis; the anti-PLA2R testing rate also differed between the two groups (P = 0.007), whereas seropositivity among tested patients did not (P = 0.494); among unevaluable patients, 67 had remission-status records before attrition, of whom 33 (49.3%) were in CR/PR at their last evaluable timepoint-not significantly different from the 66.7% (30/45) observed in evaluable patients at 12 months (P = 0.082).

### Follow-up completeness and attrition

Of the 150 PMN patients, 90 (60.0%), 68 (45.3%), 57 (38.0%), and 45 (30.0%) completed the protocol-defined assessments at 3, 6, 9, and 12 months, respectively. A baseline comparison between patients evaluable at 12 months and unevaluable patients is shown in **Table 3** (comparison by 12-month evaluability status): evaluable patients were younger (52.0 vs. 57.0 years, P = 0.039) and more often originated from the previously published cohort (P < 0.001); the anti-PLA2R testing rate was lower among evaluable patients (64.4% vs. 85.7%, P = 0.007), although seropositivity among tested patients did not differ (P = 0.494); no other baseline characteristic differed significantly. Among unevaluable patients, the CR/PR proportion at the last evaluable timepoint before attrition was 49.3% (33/67), not significantly different from the 66.7% observed in evaluable patients at 12 months (P = 0.082). Of note, the low 12-month evaluability partly reflects that newly enrolled patients had not yet reached the 12-month follow-up window rather than true loss to follow-up (see Discussion).

### Efficacy in the PMN cohort

Baseline characteristics of the PMN cohort, overall and by treatment group, are shown in **Table 3** (comparison by treatment group); within the PMN cohort, the alternative-therapy group exhibited lower eGFR [82.02 (57.45, 98.35) vs. 95.26 (78.88, 106.32) mL/min/1.73 m², P = 0.016] and higher serum albumin [31.00 (23.30, 35.00) vs. 26.10 (22.15, 31.00) g/L, P = 0.033] compared with the initial-therapy group.

Among PMN patients with evaluable data at each timepoint, the complete remission rate was 2.2% (2/90) at 3 months, 5.9% (4/68) at 6 months, 12.3% (7/57) at 9 months, and 15.6% (7/45) at 12 months; the clinical remission (CR/PR) proportions were 45.6% (41/90), 54.4% (37/68), 59.6% (34/57), and 66.7% (30/45), respectively (**Table 1**). These cross-sectional estimates are based on differing denominators (90, 68, 57, and 45 evaluable patients at 3, 6, 9, and 12 months, respectively) and should not be interpreted as longitudinal progression within the same cohort. Remission kinetics were assessed using Kaplan-Meier time-to-event analyses (**Figure 2**). The median time to achieve clinical remission (CR/PR) in the PMN cohort was 104.0 (39.0, 219.5) days (**Table 1**). Remission profiles in the PMN cohort were consistent with our prior interim analysis (16).

**Figure 2:**
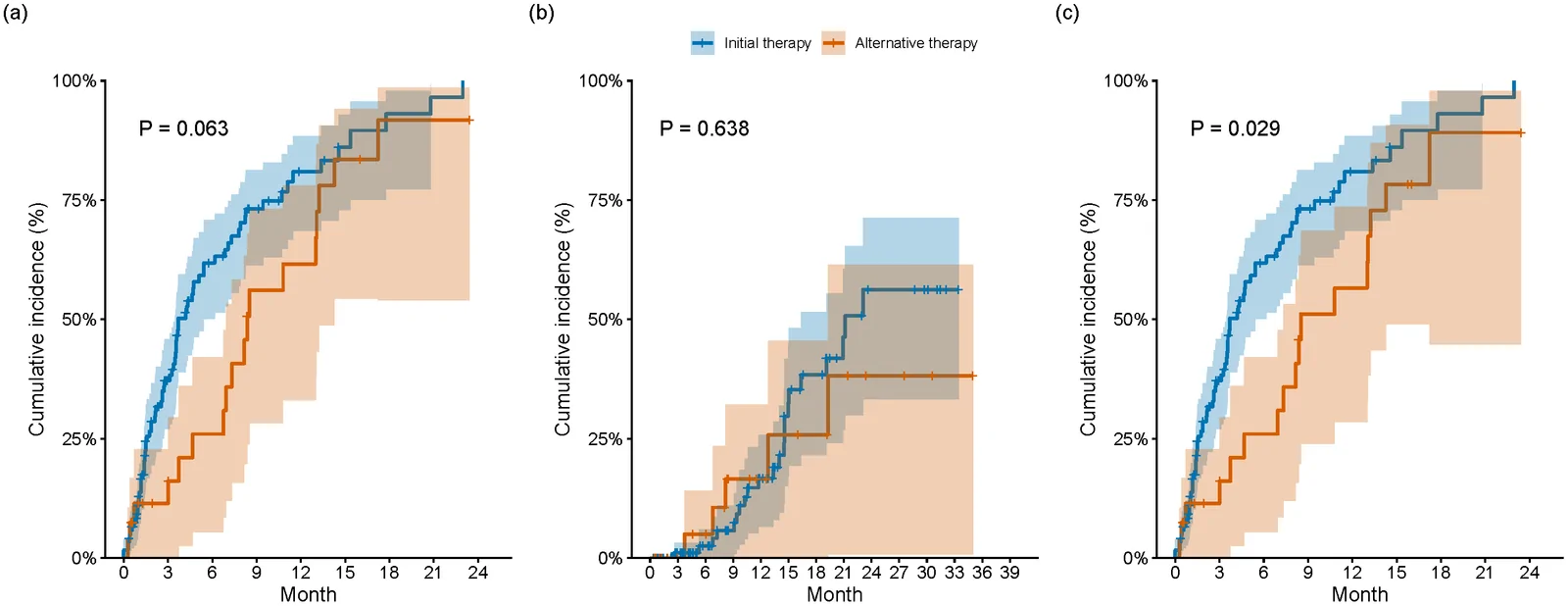
Kaplan-Meier curves for cumulative remission after ripertamab treatment in the primary membranous nephropathy (PMN) cohort. **(a)** Cumulative clinical remission (CR/PR); **(b)** Cumulative complete remission; **(c)** Cumulative partial remission. The log-rank test P-value is shown for each comparison. Orange line, alternative therapy group; blue line, initial therapy group. The timepoint-specific remission proportions reported in **Table 1** are cross-sectional estimates with differing denominators, whereas this figure presents cumulative incidence estimates based on time-to-event analysis; the two approaches are not directly comparable.

Comparing treatment-naïve (n=123) and previously treated (n=27) patients within the PMN cohort: previously treated patients achieved partial remission (median 248.0 [119.0-391.5] vs. 83.0 [36.0-163.0] days, P = 0.006) and CR/PR (median 232.5 [95.5-373.5] vs. 83.0 [36.0-163.0] days, P = 0.040) significantly more slowly, with consistent Kaplan-Meier analyses (PR: log-rank P = 0.029; CR/PR: P = 0.063); in contrast, the time to complete remission did not differ between groups (median 335.5 vs. 353.0 days, P = 0.955; log-rank P = 0.64). At 12 months, the observed CR/PR proportions were 55.6% (5/9) and 69.4% (25/36), respectively, but the previously treated estimate was based on only nine evaluable patients and should be interpreted with substantial uncertainty; the relapse rates were 35.7% (5/14) and 31.7% (20/63), respectively. Previously treated patients therefore exhibited slower remission kinetics, and the available 12-month data do not establish that longer-term outcomes are comparable between groups.

Of the PMN patients, 95 ever achieved CR/PR, of whom 77 had post-remission follow-up (the at-risk population; the remaining 18 had no post-remission follow-up). Over the follow-up period, 25 of the 77 at-risk patients (32.5%) relapsed; relapse rates in the treatment-naïve and previously treated groups were 31.7% (20/63) and 35.7% (5/14), respectively. A verification analysis restricting the time at risk to within 12 months yielded consistent results (PMN overall 20/63, 31.7%).

Of the 150 PMN patients, 119 (79.3%) underwent serum anti-PLA2R testing, of whom 81 (68.1%; 54.0% of all PMN patients) were ever positive (80 positive at first testing and 1 seroconverting from negative to positive on retesting); 39 were negative at first testing (including the aforementioned patient who later seroconverted), and 31 were never tested. The median first positive titer was 1:32 (IQR 1:10-1:32; distribution: 1:10 in 21 patients, 1:32 in 53, and 1:100 in 7). The first test was performed at a median of 0 (0-14) days from the first dose, and 100/119 patients were tested before or within 30 days after the first dose (i.e., baseline testing). Sixty-two patients had ≥2 repeat tests; among the 52 patients who were initially positive and had repeat testing, 15 (28.8%) were seronegative at the last test (immunological remission). Testing rates (76.4% vs. 92.6%, P = 0.069) and positivity rates (71.3% vs. 56.0%, P = 0.156) did not differ significantly between treatment-naïve and previously treated patients.

### Changes in laboratory indices

Longitudinal assessment of laboratory parameters demonstrated consistent improvement in both treatment groups. All patients exhibited a statistically significant reduction in 24-hour UP levels throughout the observation period (day 90 P < 0.01, days 180–360 P < 0.001). The proteinuria reduction was particularly pronounced during the first 180 days, after which the trajectory plateaued. Both groups exhibited significant and substantial proteinuria reduction throughout follow-up (all timepoints P < 0.01), with comparable magnitudes of improvement observed across treatment backgrounds (all between-group P > 0.05). Serum albumin levels increased significantly in both groups during follow-up (all timepoints P < 0.001), reflecting the restoration of glomerular permeability selective barrier function. Both groups demonstrated significant albumin recovery throughout follow-up (all timepoints P<0.001), with similar trajectories of improvement observed irrespective of prior treatment status (all between-group P>0.05). Patients with diabetes had significantly lower baseline albumin levels (P = 0.0103), although their response to ripertamab was not substantially impaired. In the PMN cohort (**Figure 3**), 24-hour UP decreased significantly from day 180 onward (P < 0.05), with no significant between-group difference in the trajectory of reduction (**Figure 3a**); serum albumin increased significantly from day 90 onward (P < 0.05), with the initial-therapy group showing a significantly greater increase at day 90 (P = 0.033) (**Figure 3b**); and eGFR increased significantly at day 90 (P = 0.006), with a greater magnitude of increase in the alternative-therapy group (**Figure 3c**).

**Figure 3:**
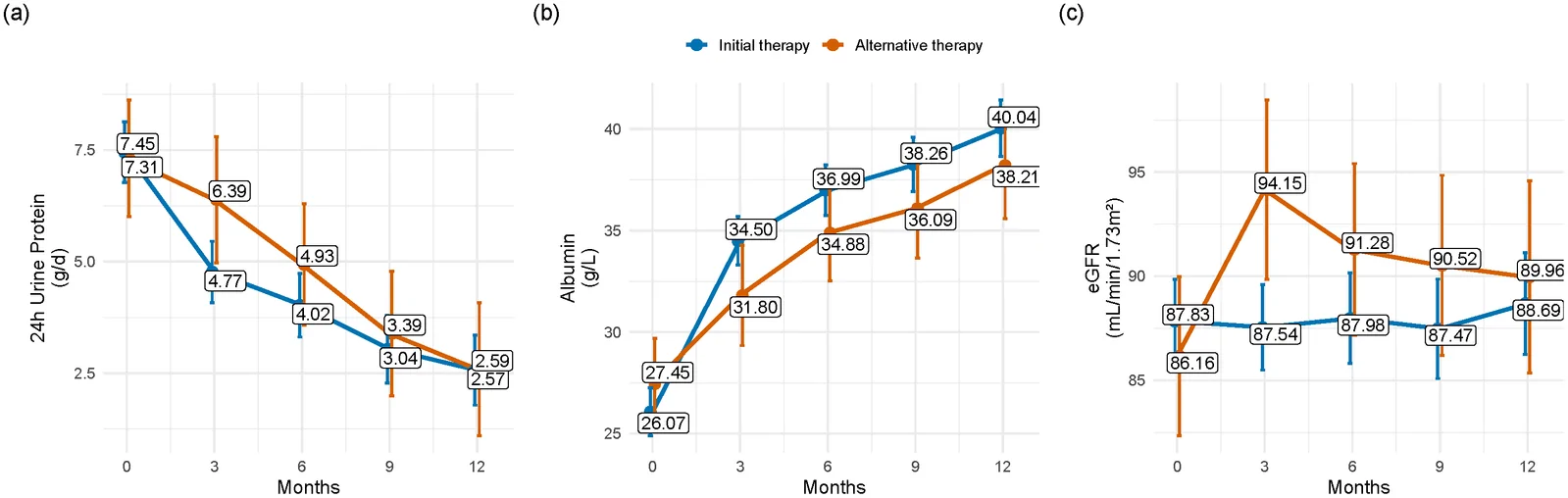
Longitudinal changes in laboratory parameters after ripertamab treatment in the PMN cohort. Data are presented as means with standard deviations. Blue line, initial therapy group; orange line, alternative therapy group. **(a)** 24-hour urinary protein; **(b)** Serum albumin; **(c)** Estimated glomerular filtration rate (eGFR).

eGFR increased significantly during the first 180 days of follow-up in both groups (P < 0.01 at 90 and 180 days), reflecting improved renal hemodynamics following proteinuria reduction. Notably, both treatment backgrounds achieved comparable eGFR preservation over the long term. This transient increase likely reflects the hemodynamic response to reduced proteinuria and improved volume status, rather than a true improvement in intrinsic renal function. After 180 days, eGFR stabilized in both groups without significant between-group differences. The overall preservation of eGFR across both groups underscores the renal safety of ripertamab in this patient population.

### Exploratory analysis of non-PMN patients

Outcomes of the 26 non-PMN patients are reported separately by individual pathological diagnosis (**Table 4**). These non-PMN data are strictly hypothesis-generating and do not constitute efficacy evidence of ripertamab in non-PMN glomerular diseases.

**Table 4:** Outcomes of non-PMN patients stratified by pathological diagnosis (n = 26).

Pathological diagnosis	n (%)	12-month CR/PR n (%)	Of which CR n (%)	Median time to remission (days)	Relapse/ever-remitted, n/N (%)
Mesangial proliferative glomerulonephritis	18 (69.2%)	7 (38.9%)	2 (11.1%)	105 [IQR 52-225]	2/7 (28.6%)
IgA nephropathy with nephrotic presentation	4 (15.4%)	2 (50.0%)	1 (25.0%)	85 [range 42-150]	0/2 (0%)
Minimal change disease	3 (11.5%)	3 (100%)	2 (66.7%)	18 [range 14-28]	0/3 (0%)
Focal segmental glomerulosclerosis	1 (3.8%)	0 (0%)	0 (0%)	—	—

Remission proportions are the numbers of patients achieving remission at 12 months divided by the total number of patients in each subgroup; no statistical comparisons were made across subgroups. The denominator for relapse is patients who had ever achieved remission. Given the small and markedly imbalanced subgroup sizes, the data in this table are strictly hypothesis-generating and do not constitute efficacy evidence of ripertamab in non-PMN glomerular diseases.

The 26 non-PMN patients exhibited heterogeneous responses that must be interpreted with substantial caution due to small sample sizes and divergent underlying mechanisms. Results are presented below by pathological category. Mesangial proliferative glomerulonephritis (n=18) achieved 12-month partial remission in 7 patients (38.9%), with complete remission in 2 (11.1%); median time to remission was 105 days (IQR 52-225), and relapse occurred in 2 of 7 responders (28.6%). IgA nephropathy with nephrotic presentation (n=4) achieved partial remission in 2 patients (50.0%) and complete remission in 1 (25.0%), with median time to remission 85 days (range 42-150) and no relapses. Minimal change disease (n=3) showed the most rapid responses, with all 3 patients (100%) achieving partial remission at 12 months including 2 with complete remission (66.7%); median time to remission was 18 days (range 14-28) with no relapses. Focal segmental glomerulosclerosis (n=1) achieved no remission.

Several key observations require cautious interpretation. First, mesangial proliferative glomerulonephritis represented the largest non-PMN subgroup, and its 38.9% partial remission rate suggests potential activity of B-cell depletion in this histopathological pattern, though the heterogeneity of underlying etiologies (including potential IgA-dominant and non-IgA variants) precludes mechanistic conclusions; notably, none of these patients had crescentic features on biopsy, distinguishing our cohort from prior studies of rapidly progressive glomerulonephritis. Second, IgA nephropathy with nephrotic-range proteinuria is uncommon and typically denotes severe disease-the 50.0% partial remission rate is consistent with prior reports of rituximab efficacy in IgA nephropathy with heavy proteinuria (17), though our sample size precludes definitive efficacy claims. Third, minimal change disease showed rapid and complete responses, which is biologically plausible given established rituximab efficacy in steroid-dependent or frequently relapsing minimal change disease (9), though our patients were treatment-naïve rather than steroid-dependent and the small sample size limits generalizability. Fourth, the single FSGS patient who failed to achieve remission aligns with the known heterogeneity of FSGS treatment responses and limited evidence for B-cell targeting in this podocytopathy (4, 10). Overall, the small sample sizes, absence of control groups, and pathological heterogeneity preclude any efficacy claims - we explicitly designate these findings as strictly hypothesis-generating and emphasize the need for dedicated studies in specific non-PMN subtypes.

### Sensitivity analyses

After excluding the 52 previously published patients, the principal efficacy signals were reproducible in the 98 independently newly enrolled PMN patients (**Supplementary Table S4**): CR/PR proportions among evaluable patients were 42.3% (22/52) at 3 months, 61.1% (22/36) at 6 months, 58.6% (17/29) at 9 months, and 58.3% (7/12) at 12 months; the median time to CR/PR was 78 (35, 163) days, not inferior to 109 (44, 304) days in the previously published cohort; and the 12-month relapse rate was 27.8% (10/36), comparable to 36.6% (15/41). Of note, only 12 newly enrolled patients were evaluable in the 12-month window (late enrollment; median potential follow-up 11.3 months), so the 12-month point estimate is imprecise; however, the early timepoints and the time-to-remission results were all consistent with the previously published cohort, and there was no indication that efficacy was driven by the previously published population.

Under extreme-scenario analysis, the 12-month CR/PR rate in the full cohort ranged from 18.8% (33/176) under the worst-case assumption to 89.2% (157/176) under the best-case assumption; in the PMN cohort, it ranged from 20.0% (30/150) to 90.0% (135/150). IPCW-adjusted CR/PR proportions at 3, 6, 9, and 12 months were 46.3% (95% CI 36.0-57.2), 57.1% (43.9-70.3), 61.4% (46.9-75.8), and 61.0% (39.4-83.6), respectively, highly consistent with the complete-case estimates (45.6%, 54.4%, 59.6%, and 66.7%). In the censoring model (**Supplementary Table S2**), age was the only significant predictor of attrition (OR 0.968 per additional year, 95% CI 0.946-0.991, P = 0.007); sex, treatment group, baseline eGFR, baseline 24-hour UP, baseline serum albumin, and remission status at the preceding timepoint were not associated with attrition (patients in remission at the preceding timepoint universally completed the next assessment-complete separation). Weight diagnostics (**Supplementary Table S3**) revealed no extreme weights (mean stabilized weight approximately 1.0, range 0.34-2.63; effective sample sizes close to the actual numbers of assessed patients; predicted probabilities of remaining under follow-up 0.39-0.83). IPCW-weighted log-rank tests yielded conclusions consistent with the unweighted analyses (CR/PR: P = 0.051 vs. 0.063; PR: P = 0.013 vs. 0.029; CR: P = 0.80 vs. 0.64).

### Adverse events

Ripertamab was well tolerated in the vast majority of patients throughout the extended follow-up period. The overall incidence of adverse events was 9.1% (16/176), with no Grade 3 or higher adverse events observed (**Table 5**). The safety profile was consistent with that reported in our prior analysis (16), in which 9.6% (5/52) of patients experienced adverse events, with no serious safety signals identified. The most common event category was infusion-related reactions (2.3%), followed by fever, rash, and facial flushing (1.7% each); upper respiratory tract infection occurred in 1.1% of patients (**Table 5**). Notably, the incidence of infusion-related reactions in our expanded cohort (2.3%) was lower than that reported in previous RTX-related studies (ranging from 22% to 80%) (6, 12), likely attributable to the standardized prophylactic premedication protocol and carefully controlled infusion rates employed across all participating centers.

**Table 5:** Adverse events associated with ripertamab during treatment and follow-up.

Adverse event	Total (n=176)	Initial therapy (n=131)	Alternative therapy (n=45)
Any adverse event, n (%)	16 (9.1)	9 (6.9)	7 (15.6)
Infusion-related reactions	4 (2.3)	2 (1.5)	2 (4.4)
Fever	3 (1.7)	1 (0.8)	2 (4.4)
Rash	3 (1.7)	2 (1.5)	1 (2.2)
Facial flushing	3 (1.7)	2 (1.5)	1 (2.2)
Pulmonary nodule	1 (0.6)	1 (0.8)	0 (0.0)
Upper respiratory tract infection	2 (1.1)	1 (0.8)	1 (2.2)
Grade ≥3 adverse events	0 (0.0)	0 (0.0)	0 (0.0)

All adverse events were Grade 1 or 2 according to the Common Terminology Criteria for Adverse Events (CTCAE) version 5.0. No serious adverse events, treatment discontinuations, or deaths related to ripertamab were observed. Infusion-related reactions were effectively minimized through prophylactic premedication with intravenous methylprednisolone (40 mg) and oral cetirizine (10 mg) prior to each infusion.

The alternative therapy group exhibited a slightly higher overall adverse event rate than the initial therapy group (13.3% vs. 6.9%), though this difference did not reach statistical significance. This trend may reflect the more refractory nature of the alternative therapy population, with prior immunosuppressive exposure potentially altering immune responsiveness and increasing susceptibility to mild adverse events. However, the absence of severe adverse events across both groups underscores the favorable tolerability of ripertamab regardless of prior treatment history.

No new safety signals emerged during the extended follow-up period. The pulmonary nodule observed in one patient was detected incidentally and was not considered related to ripertamab therapy. No treatment discontinuations or deaths attributable to ripertamab were recorded. The preservation of eGFR and the absence of nephrotoxicity further support the renal safety of ripertamab.

## Discussion

This ambispective real-world cohort study represents a substantial expansion of our prior investigation of ripertamab monotherapy in primary membranous nephropathy (16). By extending the follow-up duration of the previously published cohort from a median of 8.7 months to 18.8 months, increasing the sample size from 52 to 176 patients (PMN from 52 to 150), and incorporating a limited but clinically important subset of non-PMN immune-mediated glomerular pathologies, we have expanded the observational evidence base for ripertamab in immune-mediated glomerular diseases with proteinuria. The incremental contributions of the present study beyond the prior 52-patient analysis include: (i) multicenter expansion (52→176 patients; PMN 52→150) with extended follow-up; (ii) reproducibility of the principal efficacy signals in 98 independently newly enrolled PMN patients (**Supplementary Table S4**), excluding the possibility that the results were driven by the previously published cohort; (iii) a new within-PMN comparison of treatment-naïve versus previously treated patients (not addressed in the prior analysis); and (iv) exploratory data on non-PMN pathologies (strictly hypothesis-generating). In the pathologically homogeneous primary PMN population, ripertamab treatment was associated with clinically meaningful remission and a favorable observed tolerability profile; however, given the single-arm observational design, causality cannot be inferred.

### Remission efficacy and comparison with prior studies

The 12-month clinical remission (CR/PR) proportion among evaluable patients in the PMN cohort was 66.7% (30/45), which is consistent with our prior PMN-specific analysis (16). However, we caution that the apparent increase in remission proportions from 3 to 12 months reflects cross-sectional snapshots with markedly different denominators (90 vs. 45 patients) rather than confirmed longitudinal improvement within an identical patient subset. Longitudinal conclusions regarding remission kinetics should be drawn from the Kaplan-Meier time-to-event analyses, which appropriately handle informative censoring and varying follow-up durations. Notably, the 12-month clinical remission proportion in the PMN cohort (66.7% overall, 69.4% in initial therapy patients) remained consistent with our prior report, underscoring the reproducibility of ripertamab efficacy in this population. The inclusion of 26 non-PMN patients - representing 14.8% of the total cohort and 40.0% of the alternative therapy group - while modest in absolute number, represents an exploratory, hypothesis-generating extension beyond PMN. These findings are strictly hypothesis-generating rather than conclusive efficacy evidence, and further studies with larger non-PMN cohorts are needed to confirm efficacy in these subtypes. Among the 26 non-PMN patients, the histopathological spectrum comprised mesangial proliferative glomerulonephritis (n=18, 69.2%), IgA nephropathy with nephrotic presentation (n=4, 15.4%), minimal change disease (n=3, 11.5%), and focal segmental glomerulosclerosis (n=1, 3.8%). Notably, one patient initially classified as diabetic nephropathy was reclassified as PMN with concurrent diabetes mellitus, consistent with the established diagnostic criteria requiring biopsy confirmation and exclusion of secondary etiologies. This histopathological heterogeneity reflects the real-world clinical spectrum of immune-mediated glomerular diseases with proteinuria and underscores the importance of renal biopsy in guiding B-cell-targeted therapeutic decisions.

Several factors may explain why our observed remission rates are superior to those reported in randomized controlled trials such as MENTOR (14) and RI-CYCLO (18), yet align closely with Chinese retrospective studies (19, 20). First, RCTs employ stricter inclusion criteria, often requiring multiple 24-hour UP measurements before enrollment, which may select for more stable or treatment-resistant patients. Second, RCT populations typically exhibit higher baseline proteinuria and greater body surface area, making remission more challenging to achieve. Third, Chinese patients generally have lower body mass index and smaller body surface area than Western populations, potentially resulting in relatively greater drug exposure per unit body surface area and enhanced B-cell depletion. These pharmacokinetic considerations may contribute to the higher remission rates observed in Asian real-world cohorts.

The 12-month complete remission proportion among evaluable patients in the PMN cohort was 15.6% (7/45), which is lower than that reported by Zhang et al. (25.9%) (20) and Wang et al. (22.5%) (19) in their respective RTX-treated Chinese cohorts. Several factors may account for this discrepancy. First, loss to follow-up in real-world settings may lead to underreporting of CR, as patients who achieve complete remission may be less motivated to return for scheduled visits. In the PMN cohort, 70.0% of patients did not meet the protocol-defined 12-month assessment, partly reflecting administrative censoring of newly enrolled patients who had not yet reached the assessment window rather than true attrition (see Study Limitations), complicating the assessment of true CR rates. Second, differences in retreatment strategies: RCTs typically administer fixed reinfusion schedules regardless of clinical status (18, 21), whereas Chinese clinical practice often relies on B-cell count or anti-PLA2R antibody levels to guide retreatment (19, 22). However, due to testing availability and cost constraints, routine monitoring of these biomarkers is not uniformly performed in real-world practice, potentially resulting in less frequent retreatment and lower CR rates.

### Treatment group response patterns and relapse

The initial-therapy and alternative-therapy groups were not clinically or pathologically comparable, and between-group differences at the full-cohort level cannot be attributed solely to prior immunosuppressive exposure. Therefore, all inferences regarding the effect of prior treatment in this study are based on the within-PMN comparison (treatment-naïve n = 123 vs. previously treated n = 27).

The within-PMN analysis suggested an association between prior treatment status and remission kinetics: previously treated patients took approximately three times longer to achieve partial remission (median 232.5 vs. 83.0 days), whereas the time to complete remission and the observed 12-month remission and relapse proportions did not differ materially between groups. Nevertheless, these cross-sectional comparisons are limited, particularly because only nine previously treated patients were evaluable at 12 months. Thus, the data support delayed remission in previously treated patients but do not establish ultimately comparable longer-term outcomes. The slower response may be consistent with the biology of refractory disease-an established immune process requires more time to be reversed by B-cell depletion-rather than necessarily indicating insufficient drug activity. Because of the observational design, residual baseline differences, and small previously treated subgroup, these findings remain exploratory and support further controlled evaluation of ripertamab as a potential alternative in refractory disease. Clinically, a longer time to onset should be anticipated in previously treated patients, and premature declaration of treatment failure based on early non-response should be avoided. A slower remission in previously treated patients was also observed in the prior 52-patient analysis (median 165.0 vs. 79.0 days) (16), directionally consistent with the present findings. It should be noted that even within the PMN cohort, the two groups differed in baseline eGFR (P = 0.016) and serum albumin (P = 0.033), and the previously treated subgroup was small (n = 27); this comparison therefore remains exploratory, and its interpretation focuses on remission kinetics rather than strict causal contrast.

The relapse rate in the PMN cohort (32.5%, calculated within the at-risk population) was higher than that observed in our prior PMN-only analysis (7.7% at a median follow-up of 8.7 months) (16). Of note, the relapse rate in the prior analysis was calculated under the then-used definition (patients evaluable in the 12-month window) with shorter follow-up and without capture of relapses outside the window; the two figures are therefore not directly comparable. The extended follow-up duration of the previously published cohort (18.8 vs. 8.7 months) provides greater opportunity for relapse detection and remains the main contributor to the higher observed relapse rate. Furthermore, the alternative therapy group-comprising patients with prior treatment failure or relapse-inherently carries a higher risk of subsequent relapse. These findings underscore the importance of long-term monitoring and potential maintenance strategies in patients treated with ripertamab, particularly in those with prior immunosuppressive exposure.

### Longitudinal laboratory changes

Longitudinal laboratory assessments demonstrated consistent improvement in both treatment groups. The significant reduction in 24-hour UP and concomitant increase in serum albumin observed throughout follow-up align with prior meta-analyses of RTX-treated membranous nephropathy (23, 24). The magnitude of proteinuria reduction at 12 months and albumin elevation are clinically meaningful and comparable to those reported with RTX (23). Both groups exhibited significant reductions in proteinuria and creatinine during follow-up. While no significant between-group differences were observed in the trajectory of proteinuria reduction, a significant between-group difference in creatinine reduction was noted at day 180 (P = 0.025), with a greater reduction in the alternative therapy group. Importantly, both treatment backgrounds achieved sustained renal function preservation over the long term. The preservation of eGFR across both groups throughout the observation period supports, but does not by itself confirm, the renal safety of ripertamab and its suitability for patients with varying degrees of baseline renal function.

### Pharmacological properties and potential mechanisms

The signals observed in non-PMN glomerulopathies are strictly hypothesis-generating; their biological plausibility nonetheless warrants further investigation. Ripertamab, a recombinant human-mouse chimeric anti-CD20 IgG1 monoclonal antibody, shares the same antigen-binding variable region as rituximab but incorporates a single amino acid substitution at position 219 of the heavy chain constant region 1 (11). This structural modification results in optimized glycosylation and enhanced binding affinity to FcγRIIIa receptors on effector cells, thereby augmenting antibody-dependent cell-mediated cytotoxicity (ADCC) activity compared with the originator molecule (11, 12). These enhanced pharmacological properties may confer superior B-cell depletion in autoimmune indications, potentially contributing to the rapid responses observed in certain non-PMN pathologies. However, given the small number of non-PMN patients in our cohort (n = 26), these findings should be interpreted as hypothesis-generating rather than definitive.

The molecular mechanisms underlying ripertamab efficacy in immune-mediated glomerular diseases with proteinuria involve multifaceted immunomodulatory pathways. Through B-cell depletion, anti-CD20 therapy directly reduces the production of pathogenic autoantibodies against podocyte antigens such as PLA2R and thrombospondin type-1 domain-containing 7A (THSD7A) (13, 25). This depletion extends beyond circulating B cells to include tissue-resident B cells and short-lived plasmablasts within the kidney, attenuating immune complex deposition in the glomerular subepithelial space (14, 26). Additionally, anti-CD20 therapy disrupts B-cell-mediated antigen presentation to T cells, impairing the activation of autoreactive T helper cells and promoting regulatory T-cell expansion, which collectively restore immune tolerance (5, 15). Complement system modulation represents another critical mechanism: B-cell depletion reduces circulating levels of C5b-9 membrane attack complexes, thereby mitigating podocyte injury and preserving nephrin expression (27, 28). The shared dependence on B-cell-mediated immunity across PMN and non-PMN glomerulopathies provides a mechanistic rationale for exploring ripertamab efficacy beyond PMN, though clinical validation in larger cohorts is essential.

### Safety profile

When compared with conventional immunosuppressive regimens, ripertamab was associated with a favorable tolerability profile in this cohort. Meta-analyses have demonstrated that glucocorticoid plus cyclophosphamide achieves high remission rates but is associated with significant adverse events including severe infections, hepatic injury, malignancy, and hyperglycemia (29). Similarly, tacrolimus-based regimens, while effective, carry risks of nephrotoxicity and metabolic disturbances (30). In contrast, ripertamab was well tolerated in our cohort, with no Grade 3 or higher adverse events observed throughout the extended follow-up period. The low incidence of infusion-related reactions, effectively minimized through prophylactic premedication, further supports its suitability for outpatient administration and long-term management.

### Heterogeneity and its implications for B-cell-targeted therapy

The non-PMN findings in this study are strictly hypothesis-generating and do not constitute efficacy evidence of ripertamab in non-PMN glomerular diseases. The inclusion of non-PMN pathologies, while broadening clinical relevance, introduces mechanistic complexity that demands careful dissection. Our non-PMN cohort comprised four distinct pathological categories with divergent relationships to B-cell-driven injury. Mesangial proliferative glomerulonephritis (n=18) represents a histopathological pattern rather than a single disease entity, encompassing IgA-dominant and non-IgA variants. B cells contribute through autoantibody production and immune complex formation, though the mesangial deposition site-versus subepithelial in PMN-may alter the accessibility of pathogenic antibodies to depletion. The 38.9% partial remission rate observed in this subgroup is encouraging but requires validation in larger, etiologically characterized cohorts. IgA nephropathy with nephrotic presentation (n=4) is uncommon and denotes severe disease. B cells are central to the production of galactose-deficient IgA1 and subsequent immune complex formation (17). The 50.0% partial remission rate in this small subset is consistent with prior reports of rituximab efficacy in IgA nephropathy with heavy proteinuria, though nephrotic-range presentation may select for unusually treatment-refractory cases. Minimal change disease (n=3) showed the most rapid and complete responses. While traditionally viewed as a T-cell-mediated disorder, emerging evidence implicates B cells in producing circulating permeability factors, and the NEMO trial established rituximab efficacy in steroid-dependent or frequently relapsing minimal change disease (9). Our findings in treatment-naïve patients extend this evidence, though the small sample size precludes definitive conclusions. Focal segmental glomerulosclerosis (n=1) showed no response, aligning with the known heterogeneity of this podocytopathy and limited evidence for B-cell targeting outside specific genetic or secondary forms (4, 10). This mechanistic diversity underscores why aggregated non-PMN categorization is scientifically unsound. We report disaggregated data and urge the field to abandon lumped non-PMN categorization in favor of mechanism-stratified trials. Notably, the absence of ANCA-associated vasculitis or crescentic glomerulonephritis in our cohort with established or emerging evidence for B-cell-targeted therapy-reflects our stringent exclusion of secondary etiologies and the requirement for proteinuria as the presenting phenotype.

### Study limitations

Several limitations of this study should be acknowledged. The single-arm observational design precludes causal attribution of observed remissions to ripertamab versus disease natural history or regression to the mean, particularly given the estimated 30-40% spontaneous remission rate in primary membranous nephropathy. The absence of a concurrent control group-whether untreated, placebo, or active comparator-remains the fundamental constraint on internal validity.

That 70.0% of PMN patients did not complete the protocol-defined 12-month assessment warrants a two-part interpretation. First, a substantial proportion does not represent true attrition: newly enrolled PMN patients had a median potential follow-up of only 11.3 months and had not yet reached the 12-month assessment window (administrative censoring), and a further 16 patients had clinical follow-up beyond 12 months but assessments falling outside the protocol windows. Second, true loss to follow-up mainly resulted from routine transfer of clinically stable patients to primary or secondary facilities under China’s tiered healthcare system. Evaluable patients were younger than unevaluable patients (P = 0.039), and the pre-attrition remission proportion was numerically lower among unevaluable patients (49.3% vs. 66.7%); although the difference was not statistically significant (P = 0.082), informative censoring cannot be fully excluded. The extreme-scenario bounds (18.8%–89.2% in the full cohort and 20.0%–90.0% in the PMN cohort) faithfully delineate the residual uncertainty of the 12-month primary outcome; the IPCW-adjusted estimates were highly consistent with the complete-case estimates, suggesting that attrition did not introduce substantial bias. Additionally, we explicitly caution against interpreting the sequential CR/PR proportions at 3, 6, 9, and 12 months as longitudinal remission trajectories. Because the denominators decline substantially over time (from 90 to 45 patients), these figures represent independent cross-sectional estimates at each timepoint. Appropriate longitudinal inference relies on the Kaplan-Meier and MMRM analyses, which accommodate incomplete observations and censoring.

The heterogeneity of glomerular pathologies, while reflecting real-world clinical spectrum, introduces substantial uncertainty in non-PMN estimates. The non-PMN subgroup was dominated by mesangial proliferative glomerulonephritis, a histopathological pattern encompassing multiple distinct disease entities with divergent natural histories and treatment responses. The absence of ANCA-associated vasculitis or crescentic glomerulonephritis in our non-PMN cohort is noteworthy, as these conditions have established (vasculitis) or emerging (crescentic GN) evidence for B-cell-targeted therapy. Their exclusion reflects our stringent secondary etiology screening and the requirement for proteinuria as the presenting phenotype, which is atypical for pauci-immune diseases. We explicitly refrain from efficacy claims for specific non-PMN subtypes and present these data as hypothesis-generating only.

Additional limitations include the non-randomized treatment assignment, potential unmeasured confounding by indication, and the inability to account for traditional Chinese medicine co-interventions. The standardized premedication protocol, while reducing infusion reactions, limits generalizability to centers with different supportive care practices.

Anti-PLA2R testing in this study was driven by physician decisions in real-world practice and has three limitations: first, 31 PMN patients (20.7%) were never tested, so selection bias cannot be excluded; second, only IIFT titers without RU/mL quantification were available, limiting direct comparison with studies using quantitative assays; third, retesting intervals were not standardized, so the 28.8% last-test seronegative rate is influenced by retesting timing, is not directly comparable with trials using fixed-interval testing, and does not permit reliable estimation of the time to seroconversion. Future prospective registries should incorporate standardized serial anti-PLA2R testing (fixed timepoints, quantitative methods) into the protocol to support immunological-remission-guided retreatment strategies.

Two ripertamab dosing regimens were used in this study (375 mg/m² once weekly for 4 weeks, or 1 g per dose every two weeks for a total of 2 doses). The choice of regimen was individualized by treating physicians at each center rather than randomly assigned, and regimen information was not prospectively captured in a structured, analyzable form in this real-world registry; the current dataset therefore cannot reliably address differences in efficacy or safety between the two regimens. A head-to-head comparison of the two regimens in PMN, ideally in a randomized design, is an important direction for future research.

In summary, this expanded cohort demonstrates associations between ripertamab monotherapy and clinically meaningful remission and favorable tolerability in PMN, while providing strictly hypothesis-generating, exploratory data on select non-PMN immune-mediated glomerular diseases. Given the real-world, non-randomized, single-arm design and limited representation of non-PMN subtypes, further prospective, large-scale randomized trials are essential to confirm these findings, elucidate optimal retreatment strategies, and evaluate the long-term durability of remission across diverse histopathological subtypes.

## Conclusions

This expanded ambispective, multicenter, real-world study showed that ripertamab monotherapy was associated with clinically meaningful remission and favorable observed tolerability in adult patients with PMN and proteinuria, with a 12-month clinical remission (CR/PR) proportion of 66.7% among evaluable patients and no Grade 3 or higher adverse events. Because the study was single-arm and observational and lacked a concurrent control group, these findings demonstrate association rather than confirmed efficacy. Among patients evaluable at 12 months, the CR/PR proportions were 69.4% in treatment-naïve patients and 55.6% in previously treated patients; however, only nine previously treated patients contributed to the latter estimate, and remission occurred more slowly in this subgroup. Therefore, the data do not establish comparable longer-term outcomes and instead support further controlled investigation of ripertamab as a potential alternative in refractory PMN. Additionally, exploratory data in select non-PMN immune-mediated glomerulopathies are strictly hypothesis-generating and warrant validation in larger, prospective studies.

## Data Availability

The original contributions presented in the study are included in the article/material. Further inquiries can be directed to the corresponding author.
