# Supplementary material for: Efficacy and safety of ripertamab in immune-mediated glomerular diseases with proteinuria: an expanded real-world cohort study
Source: Front Immunol. 2026 Sep 30;17:1969321. doi: 10.3389/fimmu.2026.1969321 (PMC13661562; doi:10.3389/fimmu.2026.1969321)
Supplement: Supplementary file 1 [file Table1.docx]

## **Supplementary Tables**

**Supplementary Table S1. Remission rates of ripertamab in the full cohort (n = 176) (descriptive only)**

| **Study timepoints** | **Total (n=176)** | **Initial therapy (n=131)** | **Alternative therapy (n=45)** |
| --- | --- | --- | --- |
| **Complete remission rate, n/N (%)** |  |  |  |
| 3 months | 12/105 (11.4%) | 3/80 (3.8%) | 9/25 (36.0%) |
| 6 months | 10/80 (12.5%) | 5/58 (8.6%) | 5/22 (22.7%) |
| 9 months | 9/63 (14.3%) | 6/44 (13.6%) | 3/19 (15.8%) |
| 12 months | 9/52 (17.3%) | 6/37 (16.2%) | 3/15 (20.0%) |
| **Clinical remission (CR/PR) rate, n/N (%)** |  |  |  |
| 3 months | 53/105 (50.5%) | 40/80 (50.0%) | 13/25 (52.0%) |
| 6 months | 46/80 (57.5%) | 34/58 (58.6%) | 12/22 (54.5%) |
| 9 months | 38/63 (60.3%) | 28/44 (63.6%) | 10/19 (52.6%) |
| 12 months | 33/52 (63.5%) | 25/37 (67.6%) | 8/15 (53.3%) |
| **Time to remission (day), median [IQR]** |  |  |  |
| Complete remission | 307.00 [111.00;494.50] | 317.00 [140.00;494.50] | 213.00 [55.50;432.25] |
| Clinical remission (CR/PR) | 82.00 [35.00;210.00] | 78.00 [35.00;158.00] | 101.00 [37.75;272.25] |

Note: CR, complete remission; PR, partial remission. This full-cohort table is descriptive only; given the significant differences in pathological composition and baseline characteristics between the treatment groups (Table 1), between-group comparisons are confounded, and all efficacy inferences are based on the PMN cohort (Table 3). The denominators represent the number of patients who completed follow-up at each timepoint; these are cross-sectional estimates with differing denominators and should not be interpreted as longitudinal trajectories within a fixed cohort. Relapse in the full cohort under the corrected at-risk-population definition was 30/95 (31.6%); relapse analyses are presented for the primary efficacy population in Table 3.

**Supplementary Table S2. Censoring model**

**(pooled logistic model estimating the probability of remaining under follow-up; PMN cohort, n = 150)**

| Variable | OR | 95% CI | P value |
| --- | --- | --- | --- |
| Timepoint: 6 vs. 3 months | 1.01 | 0.54-1.91 | 0.965 |
| Timepoint: 9 vs. 3 months | 1.34 | 0.70-2.62 | 0.388 |
| Timepoint: 12 vs. 3 months | 0.84 | 0.43-1.64 | 0.600 |
| Age (per year) | 0.968 | 0.946-0.991 | 0.007 |
| Sex: male vs. female | 1.06 | 0.64-1.74 | 0.831 |
| Treatment group: initial vs. alternative | 1.00 | 0.55-1.82 | 0.987 |
| eGFR (per mL/min/1.73m²) | 0.988 | 0.976-1.000 | 0.061 |
| Baseline 24-h UP (per g/d) | 0.981 | 0.927-1.038 | 0.500 |
| Baseline albumin (per g/L) | 0.974 | 0.938-1.011 | 0.173 |
| Not in remission at preceding timepoint vs. baseline | 0.89 | 0.50-1.57 | 0.678 |
| In remission at preceding timepoint vs. baseline | Not estimable* | — | — |

Note: OR > 1 indicates a higher probability of remaining under follow-up. *Patients in remission at the preceding timepoint universally completed the next assessment (complete separation), so this coefficient is not estimable-which itself indicates that patients with better outcomes were not more likely to be lost to follow-up. Age was the only significant predictor of attrition (older patients were more likely to be lost) and was adjusted for in the IPCW analysis.

**Supplementary Table S3. Diagnostics of IPCW stabilized weights (PMN cohort)**

| Timepoint | Completed assessments, n | Median weight | Mean weight | Weight range | Max/min ratio | Effective sample size (ESS) |
| --- | --- | --- | --- | --- | --- | --- |
| 3 months | 90 | 0.99 | 1.00 | 0.72-1.40 | 1.9 | 88.5 |
| 6 months | 54 | 0.99 | 0.99 | 0.57-1.59 | 2.8 | 51.4 |
| 9 months | 45 | 0.99 | 1.01 | 0.45-1.97 | 4.4 | 40.4 |
| 12 months | 32 | 0.93 | 1.01 | 0.34-2.63 | 7.8 | 25.9 |

Note: Predicted probabilities of remaining under follow-up ranged from 0.39 to 0.83 (the positivity assumption holds). The mean weight was approximately 1 and no extreme weights were observed (maximum 2.63, far below the commonly used truncation threshold of 10), and effective sample sizes were close to the actual numbers of assessed patients, indicating efficient weights; no weight truncation was performed. "Completed assessments" in this table follows the monotone censoring framework (patients were required to have consecutively completed all prior assessments; those who skipped a window were censored at the first missed window), so the numbers at 6/9/12 months (54/45/32) differ from the cross-sectional evaluable numbers in Table 3 (68/57/45); the cross-sectional framework describes data availability at each timepoint, whereas the monotone framework is used for censoring modeling in the IPCW analysis-the two address different questions.

**Supplementary Table S4. Efficacy of ripertamab in the PMN cohort stratified by enrollment period**

| Measure | All PMN (n=150) | Previously published PMN (n=52) | Newly enrolled PMN (n=98) |
| --- | --- | --- | --- |
| **CR rate (evaluable patients)** |  |  |  |
| 3 months | 2/90 (2.2%) | 1/38 (2.6%) | 1/52 (1.9%) |
| 6 months | 4/68 (5.9%) | 0/32 (0.0%) | 4/36 (11.1%) |
| 9 months | 7/57 (12.3%) | 1/28 (3.6%) | 6/29 (20.7%) |
| 12 months | 7/45 (15.6%) | 3/33 (9.1%) | 4/12 (33.3%) |
| **CR/PR rate (evaluable patients)** |  |  |  |
| 3 months | 41/90 (45.6%) | 19/38 (50.0%) | 22/52 (42.3%) |
| 6 months | 37/68 (54.4%) | 15/32 (46.9%) | 22/36 (61.1%) |
| 9 months | 34/57 (59.6%) | 17/28 (60.7%) | 17/29 (58.6%) |
| 12 months | 30/45 (66.7%) | 23/33 (69.7%) | 7/12 (58.3%) |
| **Median time to remission (days) [IQR]** |  |  |  |
| Time to CR/PR | 104 [39;220] (n=95) | 109 [44;304] (n=42) | 78 [35;163] (n=53) |
| Time to CR | 352 [156;567] (n=95) | 536 [388;691] (n=42) | 217 [132;352] (n=53) |
| Time to PR | 105 [42;227] (n=95) | 111 [50;330] (n=42) | 78 [35;163] (n=53) |
| **12-month relapse rate (at-risk population)** | 25/77 (32.5%) | 15/41 (36.6%) | 10/36 (27.8%) |

Note: Timepoint-specific figures are cross-sectional proportions (denominator = patients evaluable within each window). The denominator for relapse is patients who had ever achieved remission and had follow-up thereafter. Median times to remission were calculated among patients who ever achieved remission under the same pipeline convention as Table 3; note that the denominator for "time to CR" includes ever-remitted patients who did not achieve CR (their last follow-up time is used), so interpretation should focus on "time to CR/PR". Only 12 newly enrolled patients were evaluable in the 12-month window (late enrollment; median potential follow-up 11.3 months), so the 12-month estimates in this column should be interpreted with caution.
